# Staphylococcal Phages Adapt to New Hosts by Extensive Attachment Site Variability

**DOI:** 10.1128/mBio.02259-21

**Published:** 2021-12-07

**Authors:** Helena Leinweber, Raphael N. Sieber, Jesper Larsen, Marc Stegger, Hanne Ingmer

**Affiliations:** a Department of Veterinary and Animal Sciences, University of Copenhagen, Copenhagen, Denmark; b Department of Bacteria, Parasites and Fungi, Statens Serum Institut, Copenhagen, Denmark; St George's, University of London; Harvard Medical School

**Keywords:** livestock MRSA, CC398, Sa3int, phage, excision, integration, ϕ13, recombinase, integrase, *S. aureus*, prophage, *attP*

## Abstract

Bacterial pathogens commonly carry prophages that express virulence factors, and human strains of Staphylococcus aureus carry Sa3int phages, which promote immune evasion. Recently, however, these phages have been found in livestock-associated, methicillin-resistant S. aureus (LA-MRSA). This is surprising, as LA-MRSA strains contain a mutated primary bacterial integration site, which likely explains why the rare integration events that do occur mostly happen at alternative locations. Using deep sequencing, we show that after initial integration at secondary sites, Sa3int phages adapt through nucleotide changes in their attachment sequences to increase homology with alternative bacterial attachment sites. Importantly, this homology significantly enhances integrations in new rounds of infections. We propose that promiscuity of the phage-encoded tyrosine recombinase is responsible for establishment of Sa3int phages in LA-MRSA. Our results demonstrate that phages can adopt extensive population heterogeneity, leading to establishment in strains lacking bona fide integration sites. Ultimately, their presence may increase virulence and zoonotic potential of pathogens with major implications for human health.

## INTRODUCTION

Staphylococcus aureus colonizes both humans and animals, and its preference is associated with the content of mobile genetic elements (1). One example is bacterial viruses, so-called prophages, of the Sa3int family. They are found in most human strains of S. aureus, where they express one or more immune evasion factors believed to facilitate human colonization as well as to promote human-to-human transmission (2, 3). In contrast, the strains of methicillin-resistant S. aureus found in livestock (LA-MRSA) commonly lack Sa3int phages (4, 5). In fact, LA-MRSA of the CC398 lineage appears to have been derived from human-associated strains which, subsequent to a jump from humans to animals, lost the Sa3int prophage (5).

Despite host preference, there is a growing number of human infections with LA-MRSA, and in 2019, they accounted for 32% of all new MRSA cases in Denmark (DANMAP, 2019). People with occupational livestock contact are most at risk (2, 6, 7), and the infections appear to be as severe as those caused by human-associated strains (8). Although human infections with LA-MRSA are considered to be the result of spillovers from livestock, there have been examples of transmissions between household members as well as into community and health care settings (2, 3, 7). Importantly, such transfer events were associated with LA-MRSA strains carrying prophages of the Sa3int family (2, 3, 7, 9). As 95% of tested Danish pig herds are positive for LA-MRSA (DANMAP, 2019), establishment of Sa3int phages in these strains may pose an increased risk of community spread of LA-MRSA strains.

Integration of Sa3int phages in S. aureus occurs through orientation-specific recombination between identical 14-bp phage and bacterial core attachment sequences (*attP* and *attB*, respectively) and is mediated by a phage-encoded tyrosine recombinase, the integrase Int (10, 11). In livestock strains, the sequence corresponding to *attB* has two nucleotide changes (underlined): 5′-TGTATCCGAATTGG-3′ (*attB_LA_*). These substitutions do not alter the amino acid sequence of the β-hemolysin encoded by *hlb* in which *attB* is located but significantly decrease the ability of Sa3int phages to insert at this location by approximately 2 log (12). Accordingly, in LA-MRSA strains, Sa3int prophages are mostly located at alternative integration sites with variable positions in the bacterial genome but occasionally also in *attB_LA_* (2, 12–15).

S. aureus has on several occasions demonstrated its ability to alter its preference for human or animal hosts. In general, such “host jumps” are thought to occur when infections of less preferred hosts are followed by host adaptation, ultimately leading to colonization (2, 16). Host adaptation often involves acquisition or loss of mobile genetic elements, including prophages (1). However, little is known of the molecular events involved in the process. Using massive parallel sequencing, we examined the fate of Sa3int phages interacting with a S. aureus strain carrying the *attB_LA_* of LA-MRSA. We found that initial, rare integration events at alternative integration sites located across the bacterial genome led to phage populations with highly variable *attP* sequences, of which a greater part increased resemblance to the bacterial attachment sequence. Importantly, infections of naive strains carrying the *attB_LA_* site with such phage pools resulted in increased phage integration. Our results explain how Sa3int phages, by adapting their *attP* sequence to alternative integration sites in the LA-MRSA genome, can establish in these strains that ultimately may be more successful at colonizing and infecting humans and disseminate in the human population.

## RESULTS

### Sa3int phages are adapting to alternative *attB* sites of LA-MRSA CC398.

**FIG 1:**
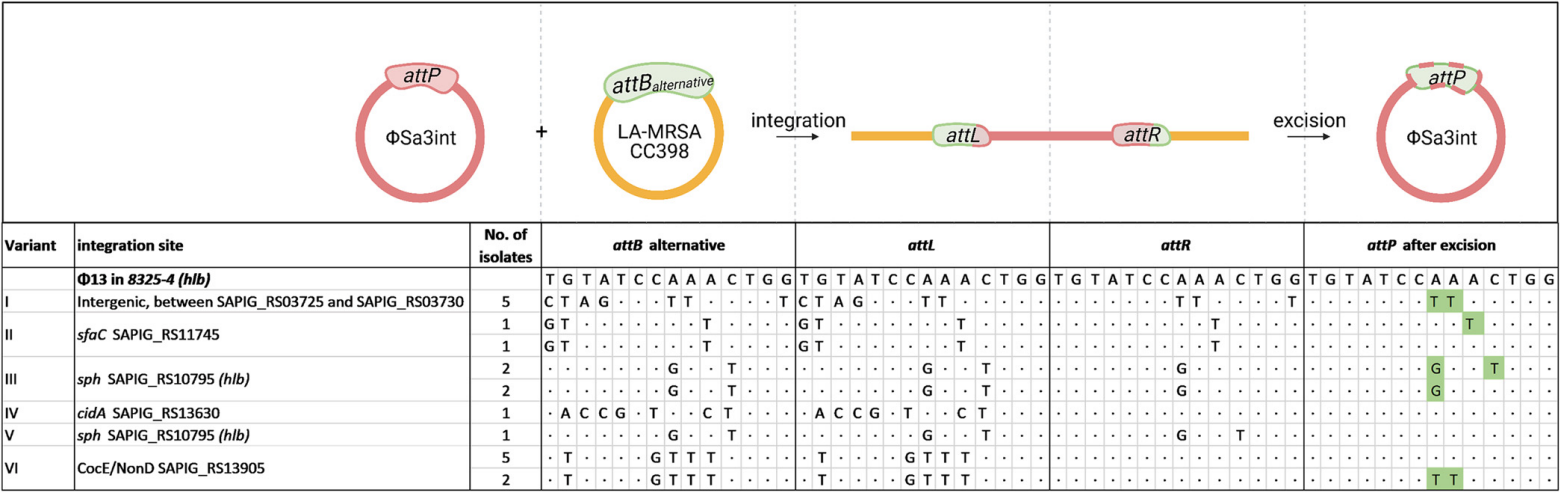
Sa3int phages excised from LA-MRSA CC398 strains display *attP* sequence variability. Visible nucleotides indicate mismatches between the *attB* site in S. aureus 8325-4 and the *attB* site deduced for each isolate; a dot indicates nucleotide conservation. Green highlighting indicates changes in *attP* that lead to identity with the bacterial attachment site. The threshold for variant calling was set to 50%. The schematic drawing illustrates the formation of *attL* and *attR* upon phage integration into an alternative *attB* site (indicated by red- and green-dashed *attP)*. After excision, most *attP* sites have adapted to the alternative *attB* site (green).

In a recent study, 20 LA-MRSA CC398 strains from pigs and humans in Denmark were isolated and found to contain Sa3int prophages. In these strains, the prophages were located at one of five different genomic locations (variants I to VI) (2), and the respective variants were isolated from the same household and are epidemiologically related. The 14-bp primary bacterial integration site in *hlb* carried two nucleotide mismatches (designated *attB_LA_*) compared to the one found in human strains in other studies of LA-MRSA strains (12, 14, 15). We determined the sequences flanking the prophage (*attL* and *attR*) in the LA-MRSA CC398 genomes, and through comparisons with strains that lack the prophage, we deduced the corresponding *attB* sequences (Fig. 1). In all cases except one (variant V), the *attL* sequences differed from *attR*. This indicates nonmatching *attB* and *attP* sites, as otherwise *attR* and *attL* would be identical, as seen with the original *attB* site in *hlb* of S. aureus 8325-4. Searching a 300-bp area flanking the alternative *attB* site did not reveal any conserved motifs.

To examine if mismatches between *attL* and *attR* affected excision of the prophage, we induced the lysogens with mitomycin and observed that in all strains the phages could be excised. From the resulting phages, we determined the *attP* sequences using PCR amplification and Sanger sequencing (Fig. 1). For eight phages (one isolate each of variants II, IV, and V and five isolates of variant VI), the *attP* sequences were identical to that of the model Sa3int phage ϕ13 (10), showing that in these cases integration in the variant *attB* sites did not affect the *attP* sequence of the excised phage. In the remaining 12 phages, however, mutations had arisen in the phage *attP* sequences. Importantly, in all cases, the changes increased the sequence similarity between *attP* and the alternative *attB* site of the livestock-associated strains, as indicated in Fig. 1. These results suggest that Sa3int phages may be promiscuous with respect to both integration and excision and that integration of prophages at alternative bacterial attachment sites may alter the phage in such a way that its *attP* sequence bares greater resemblance to alternative *attB* sequences.

### Phage integration at multiple locations in a model strain carrying *attB_LA_*.

With the aim of investigating how phage heterogeneity arises we employed a derivative of S. aureus NCTC8325-4, designated S. aureus 8325-4attBmut, which contains 2-bp point mutations in *hlb* to create the *attB_LA_* of the LA-MRSA CC398 lineage (12). With this strain, we performed liquid infection with ϕ13kan^R^, a derivative of the Sa3int phage ϕ13 that encodes staphylokinase (*sak*) but in which the immune evasion virulence genes *scn* and *chp* are replaced by the kanamycin resistance cassette *aphA3* (12).

**FIG 2:**
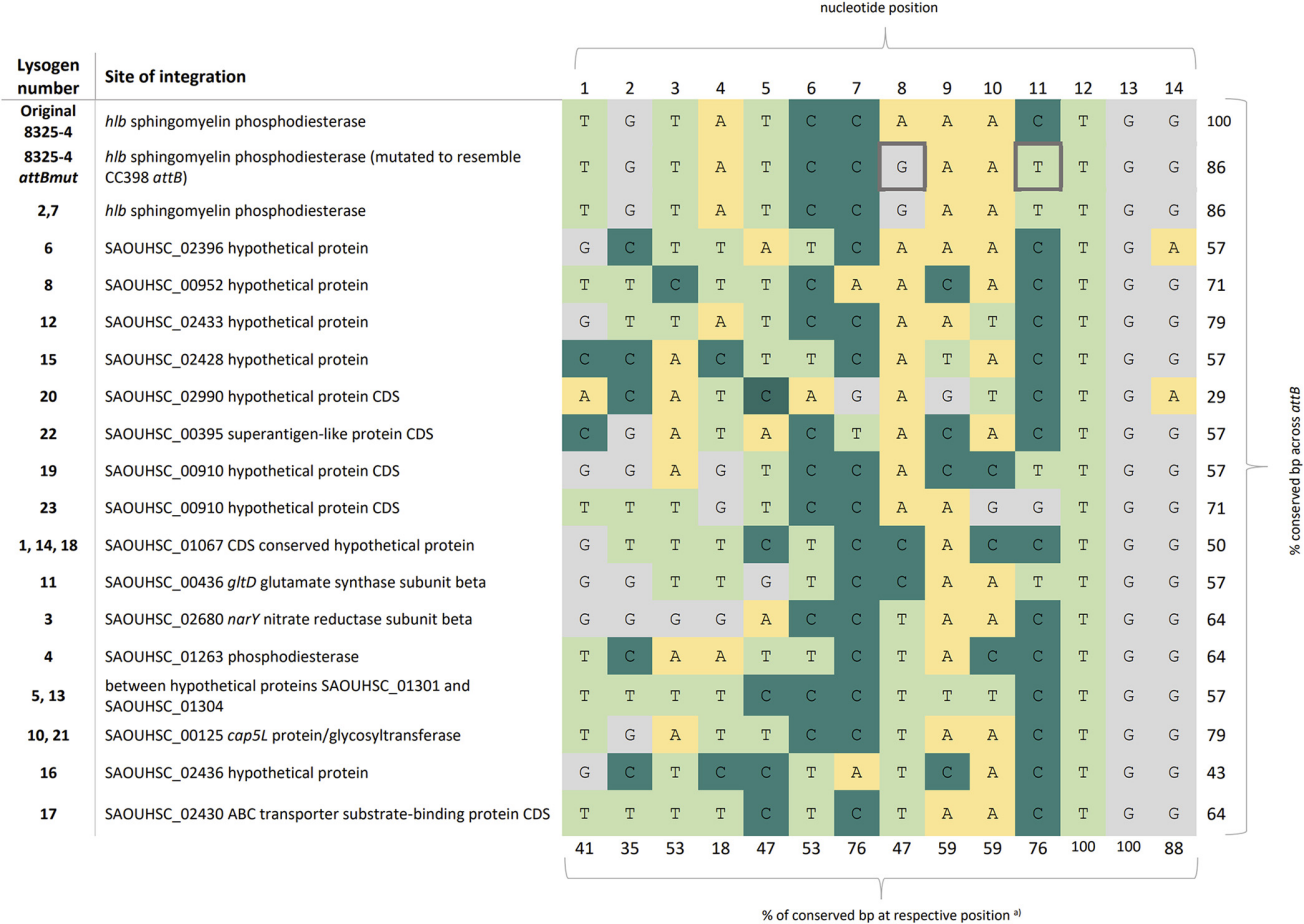
Alternative integration sites of ϕ13kan^R^ in S. aureus 8325-4attBmut. The core *attB* sites are presented by color coding of the different base pairs (yellow, A; dark green, C; light green, T; gray, G). The mutated base pairs in *hlb* representing *attB_LA_* in the recipient strain are indicated by a bold frame. a) the percentages in the bottom row correspond to the proportions of conserved nucleotides in the 17 alternative *attB* sites found in the 22 lysogens with respect to the original *attB* in 8325-4.

From eight independent lysogenization experiments, we selected 22 lysogens as being resistant to kanamycin. Alternative integration sites were confirmed for 20 of the lysogens by PCR (*hlb*^+^ *sak*^+^), and two lysogens harbored the phage in the mutated *hlb* site (*hlb*^−^ *sak^+^*) (Fig. S1). The 22 isolates were whole-genome sequenced, and analysis revealed 17 different integration sites for ϕ13kan^R^ in S. aureus 8325-4attBmut that were widely distributed across the bacterial chromosome (Fig. S2) and with the *attB* sequences listed in Fig. 2. The integrations occurred in both noncoding and coding regions and were independent of transcriptional orientation.

10.1128/mBio.02259-21.1 FIG S1 Gel electrophoresis after PCR for *hlb* (top) and *sak* (bottom). The pictures were taken from different rounds of PCR amplification (as indicated by the white cuts between the pictures) and therefore are not completely aligned. Ladder is a 1kb+ GeneRuler from Thermo Fisher Scientific. Lane numbers indicate lysogen numbers. Lanes marked with an “x” either are empty or contain an unrelated sample. Download FIG S1, TIF file, 0.6 MB. Copyright © 2021 Leinweber et al. 2021 Leinweber et al. https://creativecommons.org/licenses/by/4.0/ This content is distributed under the terms of the Creative Commons Attribution 4.0 International license.

10.1128/mBio.02259-21.2 FIG S2 Location of the alternative integration sites of ϕ13 indicated in the reference chromosome of S. aureus NCTC8325 (NC_007795.1). The arrows indicate the translation direction of the respective gene. The triangle on top exemplifies the phage, with integration direction shown by “L,” indicating *attL*, and “R,” indicating *attR* (which is the site where the integrase is located). The figure was generated using Geneious Prime 2021.1.1 and Microsoft PowerPoint. Download FIG S2, TIF file, 0.2 MB. Copyright © 2021 Leinweber et al. 2021 Leinweber et al. https://creativecommons.org/licenses/by/4.0/ This content is distributed under the terms of the Creative Commons Attribution 4.0 International license.

When the 14-bp sequences of all alternative *attB* sites were compared (Fig. 2), they showed 29 to 86% homology compared to the original *attB* core sequence in the *hlb* gene. However, the last three base pairs (5′-TGG-3′) were highly conserved, being present in 20 of 22 *attB* sites, with lysogens 6 and 20 being the exceptions. The nucleotides G at position 8 and T at position 11, signifying *attB_LA_* compared to *attB*, were not found in the same combination in any of the 17 *attB* sequences. Based on the conserved base pairs between the alternative *attB* sites, we searched the chromosome of S. aureus NCTC8325 for the presence of 5′-NNNNNNCWNNCTGG-3′ (where W = A or T) and obtained more than 700 hits. Thus, there appears to be a multitude of potential integration sites in the staphylococcal genome.

Three of the alternative *attB* locations were observed as integration sites in lysogens obtained in independent lysogenization rounds, i.e., the SAOUHSC_01067 coding sequence (CDS) conserved hypothetical protein (lysogens 1, 14, and 18), the intergenic region between open reading frames encoding the hypothetical proteins SAOUHSC_01301 and SAOUHSC_01304 (lysogens 5 and 13), and the SAOUHSC_00125 *cap5L* protein/glycosyltransferase (lysogens 10 and 21). As clonality can be excluded, these integration events show that there is some preference in selection of integration site when the bona fide *attB* sequence is mutated. However, when we screened the 300-bp flanking regions of the alternative *attB* sites in S. aureus 8325-4attBut, we found no common patterns in terms of sequence composition or distance of inverted repeats relative to the alternative *attB* core sequences (Fig. S3 and S4). Thus, it is still unclear why some integration sites are preferred over others.

10.1128/mBio.02259-21.3 FIG S3 Inverted and direct repeats of >10 bp found in the 300-bp flanking sequence of the 17 alternative *attB* sites in S. aureus 8325-4attBmut. Identical sequences are highlighted in the same color. The red box in the center marks the 14-bp *attB* sequence. The search was carried out with repeat finder in Geneious Prime 2021.1.1. Download FIG S3, TIF file, 2.5 MB. Copyright © 2021 Leinweber et al. 2021 Leinweber et al. https://creativecommons.org/licenses/by/4.0/ This content is distributed under the terms of the Creative Commons Attribution 4.0 International license.

10.1128/mBio.02259-21.4 FIG S4 Sequence logo of a sequence alignment of 300 bp flanking the 17 alternative *attB* sites of S. aureus 8325-4attBmut. The 14-bp *attB* site is highlighted with a square box. Each stack of letters represents one position in the DNA sequence. The height of the stack indicates the sequence conservation at the respective position, and the size of the letter within the stack represents the relative frequency of each nucleotide at this position. The logo was created using weblogo.threeplusone.com. Download FIG S4, TIF file, 0.6 MB. Copyright © 2021 Leinweber et al. 2021 Leinweber et al. https://creativecommons.org/licenses/by/4.0/ This content is distributed under the terms of the Creative Commons Attribution 4.0 International license.

### Phage evolution following excision from alternative integration sites.

In agreement with our observations for Sa3int phages in livestock-associated strains, we found that mitomycin C induced ϕ13kan^R^ from all lysogens established in the 8325-4attBmut strain with the number of phage particles varying between 5 × 10^3^ PFU/mL and 4 × 10^6^ PFU/mL (Fig. S5). This represents up to a 1,000-fold decrease in induction efficacy compared to the 6 × 10^6^ PFU/mL obtained when the phage was induced from its integration site in the nonmutated *attB* of S. aureus 8325-4 (8325-4phi13kan^R^ control). Spontaneous phage release was also detected for many of the lysogens, ranging from 2 × 10^1^ to 3 × 10^3^ PFU/mL, compared to 1.0 × 10^4^ PFU/mL for the 8325-4phi13kan^R^ control (Fig. S5).

10.1128/mBio.02259-21.5 FIG S5 Excision of ϕ13kanR from alternative integration sites. The number of PFU/mL observed from the 22 lysogens after spontaneous release (light green bars) or when induced with 2 μg/mL mitomycin C (dark green bars) is shown. The starting cell count (CFU/mL) is also shown (grey diamonds) to exclude differences in PFU due to varying inoculum sizes. Error bars represent standard deviations for three biological repeats. Download FIG S5, TIF file, 0.4 MB. Copyright © 2021 Leinweber et al. 2021 Leinweber et al. https://creativecommons.org/licenses/by/4.0/ This content is distributed under the terms of the Creative Commons Attribution 4.0 International license.

To examine the integration and excision process of ϕ13kan^R^ at the alternative integration sites, we determined the *attL* and *attR* sequences from the genome sequences of the lysogens and deduced the alternative *attB* sites by comparing with sequences prior to integration of the phage. In addition, we determined the *attP* sequences by induction of the lysogens and amplicon sequencing of PCR products obtained on phage lysate with primers spanning *attP* (sequencing depth range, 10,000 to 180,000; average, 100,000).

**FIG 3:**
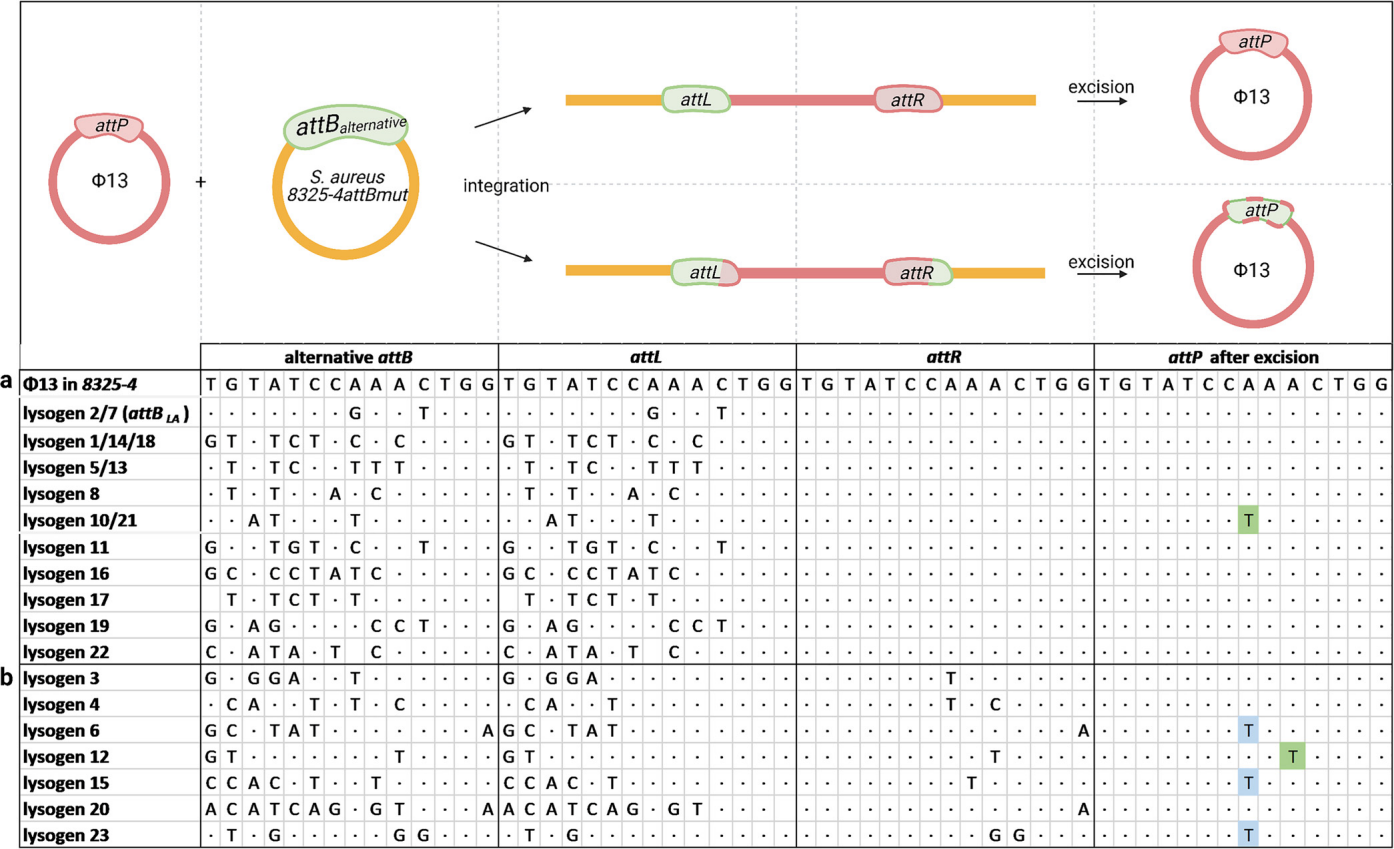
Sequence variability is recreated by integration of ϕ13kan^R^ in 8325-4attBmut. Visible nucleotides indicate mismatches between the *attB* site in S. aureus 8325-4 and the alternative *attB* site of each lysogen; a dot indicates nucleotide conservation. Green highlighting indicates changes in the *attP* site that mimic the *attB* site. Blue highlighting indicates other changes in the *attP* site. The threshold for variant calling was set to 50%. (a) The upper part of the table shows lysogens where *attL* matches *attB*, and *attR* matches *attP*; (b) the lower part shows the lysogens where parts of *attL* and *attR* both match *attB* and *attP*. The schematic drawing illustrates the formation of *attL* and *attR* upon phage integration into an alternative *attB* site (indicated by red- and green-dashed *attP*). After excision, *attP* either is unchanged or has adapted to the alternative *attB* sites.

**FIG 4:**
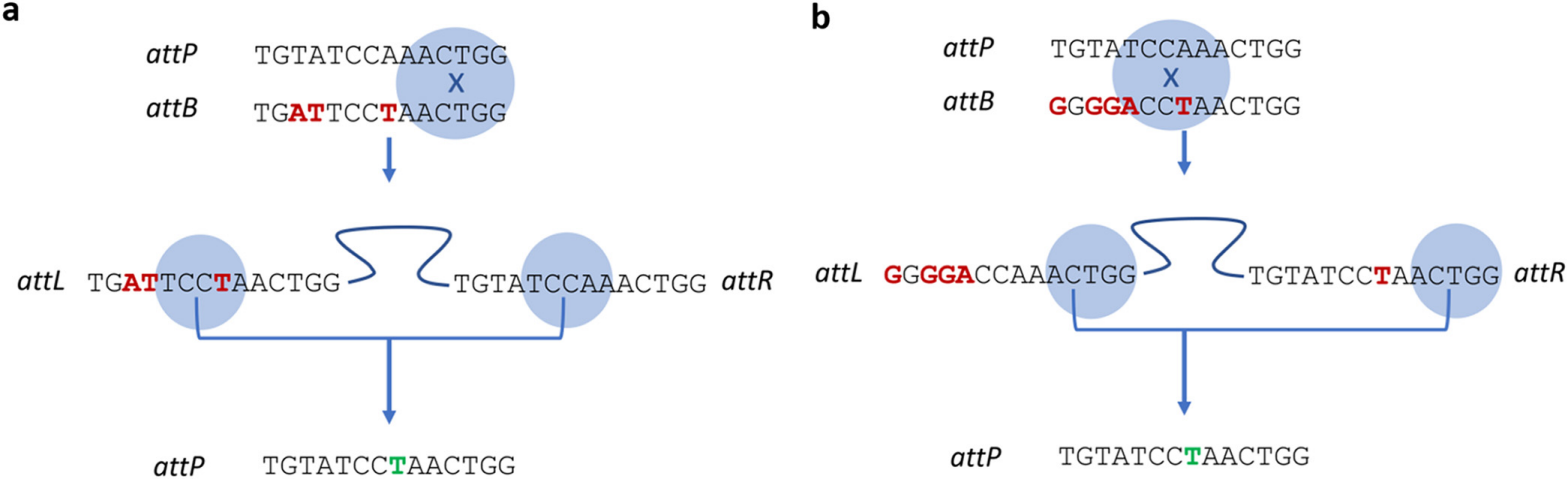
Schematic process of chromosomal recombination between *attP* and *attB*. Schematics show the integration event happening toward the 3′ end of the *att* sites and excision happening toward the center of the *att* sites (a) (example of lysogens 10 and 21) and vice versa (b) (example of lysogen 3).

For the majority of the lysogens (Fig. 3a), *attL* was identical to *attB*, and *attR* was identical to *attP*, as can be observed by the pattern of letters (representing nonmatching nucleotides) or dots (representing conserved nucleotides). For these lysogens, the integration crossover likely occurred at the 5′-TGG-3′ (Fig. 4a). For the remaining lysogens (Fig. 3b), both *attL* and *attR* displayed sequences matching the alternative *attB* site, with *attL* matching the 5′ end and *attR* the 3′ end. In these cases, the integration crossover events may have occurred at variable positions within the core sequences (Fig. 4b).

**FIG 5:**
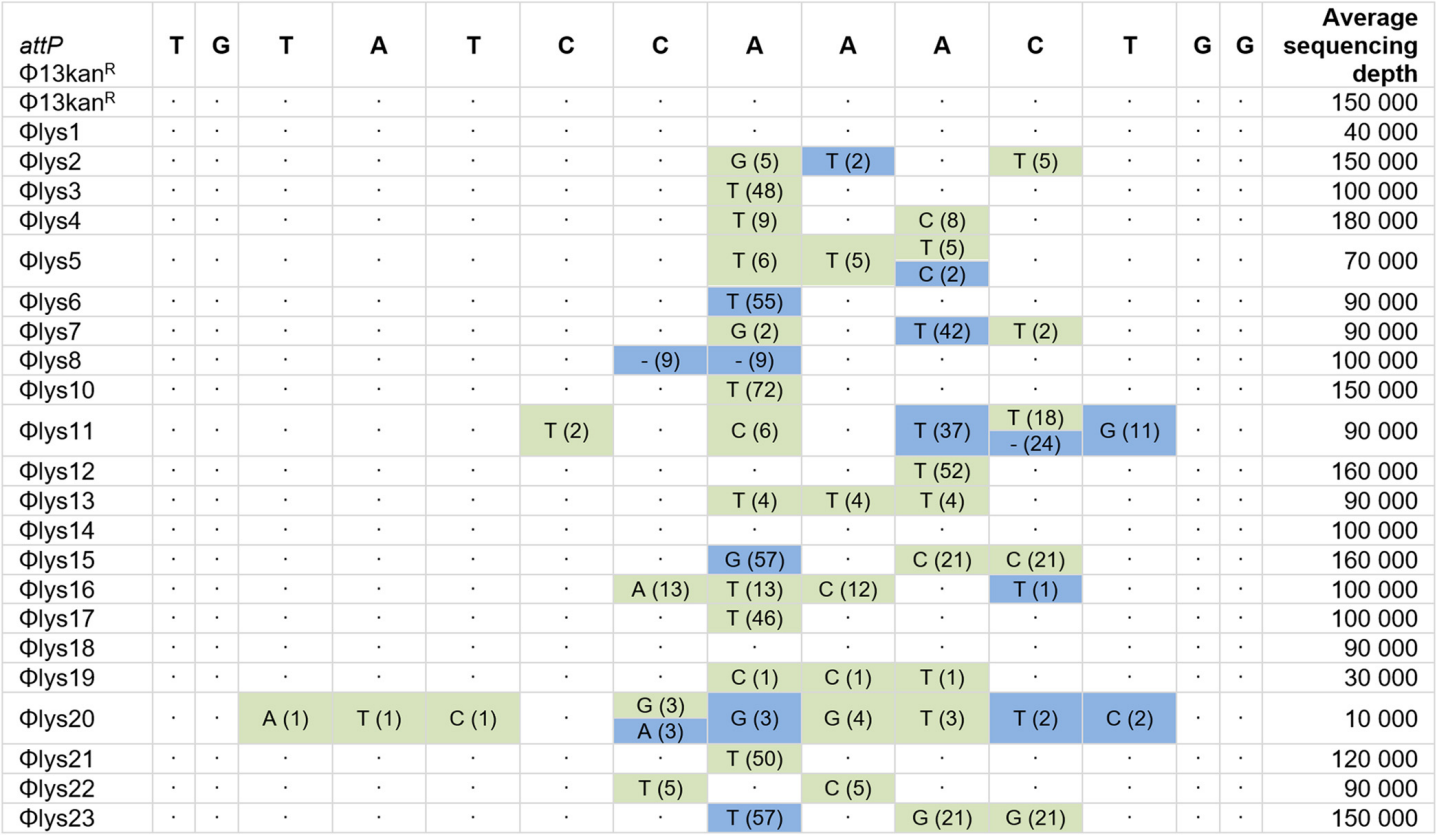
Variability in *attP* in phages excised from alternative bacterial integration sites. Variant nucleotides and respective frequencies (in percent) of the *attP* sequences after excision of the phages were determined by amplicon sequencing. The green shading indicates changes in the *attP* site that mimic the respective alternative *attB* site. Blue shading indicates other changes at this position. Dashes indicate that no nucleotide was detected at this position. Dots indicate conservation compared to *attP* in ϕ13kan^R^, the sequence of which is in the first row. Note that for ϕ13kan^R^, ϕlys1, ϕlys14, and ϕlys18, no variants with frequencies of >1% were detected across the entire *attP* sequence.

When assessing *attP* by amplicon sequencing, we observed remarkable sequence variation at single nucleotide positions in more than 40% of the phage populations obtained from 9 of the lysogens (Fig. 5). When comparing these changes to the sequence of the bacterial integration site from which the phage was derived, we saw that in five instances (lysogens 3, 10, 12, 17, and 21), the excised phages displayed adaptation to the alternative *attB* site by adopting a nucleotide of the alternative *attB* sequence (Fig. 5). Phages from lysogens 6, 7, 15, and 23 also displayed single nucleotide substitutions in *attP* but without matching the alternative *attB* sequences. These may result from mismatch repair or DNA replication after prophage excision, as has been suggested for E. coli phage P1 (17).

The adaptability of the phage to the alternative integration sites was even more pronounced when all sequence variation of >1% was scored (Fig. 5). Importantly, most of the excised phage pools contained variants with sequence changes adopting the nucleotides of the alternative *attB* sequences, and multiple sequence variations occurred within the individual pools (Fig. 5, green). Notable exceptions were lysogens 1, 14, and 18, for which no variants at >1% were observed. In these lysogens, ϕ13kan^R^ had independently integrated in the same *attB* site, and despite 7 mismatches with the 14-bp *attB* sequence from 8325-4, resolution to the original *attP* sequence occurred with the same precision as seen when ϕ13kan^R^ was excised from *attB* of 8325-4phi13kan^R^. In summary, our results demonstrate that excision of ϕ13kan^R^ from alternative integration sites leads to evolutionary adaptation of the phage to the bacterium by increasing the number of *attP* nucleotides matching the alternative *attB* sequences.

### Phage adaptation to alternative *attB* sites.

**FIG 6:**
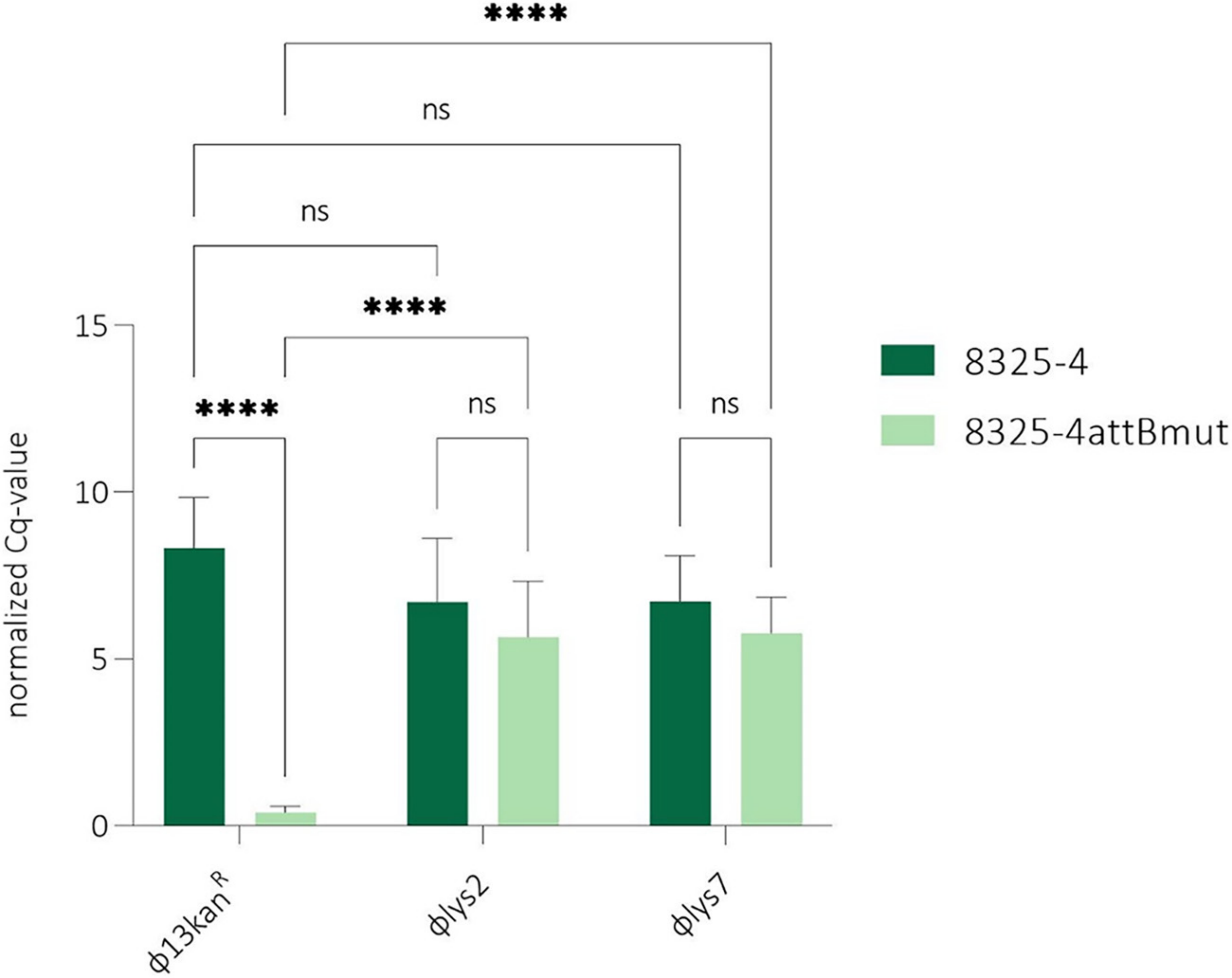
Phage adaptation enhances integration into alternative bacterial integration sites. Phage integration was detected by qPCR. The normalized *C_q_* value (calculated as 2*^Cq^*^(^*^pta^*^)−^*^Cq^*^(^*^hlb^*^)^) normalizes the cycle number of the gene of interest to the reference gene *pta*. Primers identified integration in *hlb* (*attB* or *attB_LA_*) for infection of 8325-4 (dark green) or 8325-4attBmut (light green) with either ϕ13kan^R^ or the adapted phages ϕlys2 and ϕlys7, excised from *attB_LA_*. Statistical analysis was carried out in GraphPad Prism 9.1.0, using two-way analysis of variance (ANOVA). ns, *P* > 0.05; ****, *P* < 0.0001. Error bars represent standard deviations for three biological replicates with three technical replicates.

After observing that induction of phages at alternative integration sites led to mutated phage populations with increased base pair matches between *attP* and the alternative *attB* sites or *attB_LA_*, we wondered whether these phages, in comparison to the original ϕ13kan^R^, had increased preference for such sites in a new infection cycle. To address this, we quantified integration by qPCR with primer pairs covering *attR*. We examined phage pools obtained from lysogen 2 and 7 (designated ϕlys2 and ϕlys7) excised from *attB_LA_* and compared them to the original ϕ13kan^R^ with respect to integration in either 8325-4 or 8325-4attBmut (Fig. 6). As expected, we found that for the wild-type, homogeneous ϕ13kan^R^, there was much less integration in *attB_LA_* than *attB* that matches the *attP* sequence. In contrast, this difference was essentially eliminated for the ϕlys2 and ϕlys7 phage pools. The still rather high integration frequency at the original *attB* is probably because the phage pool likely contains phages with the original *attP* sequence, which continues to integrate at *attB*. Further, the mutations in these pools significantly increased the integration frequency in 8325-4attBmut compared to ϕ13kan^R^ with the original *attP* site. Our results show that a single round of integration and excision dramatically increases the preference of the phage for an alternative or mutated attachment site.

## DISCUSSION

Sa3int prophages encode immune evasion factors and are found in most human strains of S. aureus (18, 19). In contrast, LA-MRSA commonly lacks Sa3int phages (5), but when present, they increase the risk of transmission between household members and the community (2, 3). The primary integration site for Sa3int phages is naturally mutated in livestock-associated strains, and so integration is infrequent and occurs at alternative sites (12–15). Our whole-genome analysis of S. aureus lysogens with ϕ13 integrated at alternative sites showed that recombination between nonmatching *attB* and *attP* sites leads to mismatches between the *attL* and *attR* sequences. Intriguingly, induction of these lysogens resulted in phage populations that were heterogeneous with respect to their *attP* sequences and that had changes that increased identity to the alternative bacterial integration sites (Fig. 3 and 5). Importantly, we could show that in two cases the nucleotide changes in *attP* increased phage integration into the naive 8325-4attBmut strain in a new round of infection (Fig. 6). As Sa3int prophages are spontaneously released from alternative integration sites, environmental stimuli are not necessary for dissemination of the phages. Thus, rounds of excision and integration can take place with the potential for adaptation of *attP* in each round.

When examining Sa3int prophages from outbreak strains of LA-MRSA (2), we observed a greater number of adaptive changes in the *attP* sites of the excised phages than in our model 8325-4attBmut strain. This suggests that adapted phages have been circulating in the LA-MRSA CC398 population. This notion is supported by a study of the Sa3int phage P282 from an S. aureus CC398 strain, where the *attP* sequence can be deduced to be identical to *attB_LA_* (14), although this was not noted by the authors. Also, reanalysis of genome sequence data of Sa3int-prophages in MRSA CC398 isolates from hospital patients in Germany (15) revealed that in 10 of 15 lysogens, the *attL* and *attR* sequences were identical to *attB_LA_* (Table S3), indicating that the prophages have adapted to the livestock-associated strains. This raises the question of where these phage adaptations occur. In the farm environment, humans are exposed to LA-MRSA on a continuous basis, and as about one in three humans is naturally colonized with S. aureus strains containing Sa3int phages, the livestock-associated strains are exposed to the phage. Once established as a prophage in a LA-MRSA, Sa3int phages are released and, if adapted, will integrate more effectively than the original phage into the LA-MRSA population. This in turn will lead to increased transmission from human to human and potentially be the cause of severe and difficult-to-treat infections.

10.1128/mBio.02259-21.8 TABLE S3 Alternative integration sites extracted from previous studies. In the study by Kraushaar et al. (14), the authors stated *attB* sequences that referred to *attR*; hence, the information is given in grey font but kept to show the alternative sites. For the study by Goerke et al. (35), no sequence data were available. Download Table S3, PDF file, 0.3 MB. Copyright © 2021 Leinweber et al. 2021 Leinweber et al. https://creativecommons.org/licenses/by/4.0/ This content is distributed under the terms of the Creative Commons Attribution 4.0 International license.

Integration at secondary sites has been observed for phages other than Sa3int phages when the primary integration site is absent or mutated (20–23). Excision of phage λ from such a site resulted in substitutions in *attP* (24, 25), and the authors stated that in P2, the new *attP* region contained DNA from *attR* (26, 27), but neither study showed increased integration in a new infection cycle. Similar to ϕ13, these phages encode tyrosine recombinases (11, 22). This family of recombinases catalyzes recombination between substrates with limited sequence identity (28). We propose that the adaptive behavior of Sa3int phages is dependent on this promiscuity. As tyrosine-type recombinases are employed by a number of staphylococcal phages that encode virulence factors (29), our results may provide a more general explanation for how phages adapt to new bacterial strains and thereby enable the host jumps that are regularly observed for S. aureus (1).

In summary, we have shown that rapid adaptation of S. aureus prophages to alternative integrations sites is mediated through nucleotide changes of the phage *attP* site and that excision from alternative sites leads to extensive variety in the phage pool. This facilitates phage integration in LA strains where the preferred *attB* site is absent. We suspect that the promiscuity of the phage-encoded tyrosine recombinase is responsible for this evolutionary mechanism and expect further research in this field to reveal this behavior also for other tyrosine recombinases.

## MATERIALS AND METHODS

### Strains and media.

Phage-cured S. aureus 8325-4 (30) and its mutant 8325-4ϕ13attBmut (12) (here termed 8325-4attBmut) containing the 2-bp variation in *hlb* were used as recipients and indicator strains for ϕ13kan^R^. Twenty LA S. aureus strains harboring Sa3int phages were analyzed for their *attR* and *attL* composition (2). S. aureus S0385 (GenBank accession no. NC_017333) was used as a reference strain for analysis of sequencing data of the LA strains. The prophage ϕ13kan^R^ carries the kanamycin resistance cassette *aphA3*, which replaces the virulence genes *scn* and *chp* and was obtained by induction of 8325-4phi13kan^R^ (12). A full strain list is provided in Table S1. Strains were grown in tryptone soy broth (TSB) (CM0876; Oxoid) and tryptone soy agar (TSA) (CM0131; Oxoid). Top agar for the overlay assays was 0.2 mL TSA/mL TSB. Kanamycin (30 μg/mL) and sheep blood agar (5%) were used to select for lysogens.

10.1128/mBio.02259-21.6 TABLE S1 S. aureus strains used in this study. Download Table S1, PDF file, 0.3 MB. Copyright © 2021 Leinweber et al. 2021 Leinweber et al. https://creativecommons.org/licenses/by/4.0/ This content is distributed under the terms of the Creative Commons Attribution 4.0 International license.

### Lysogenization assay.

To obtain the phage stock, 8325-4phi13kan^R^ was grown to late exponential phase (37°C, 200 rpm; optical density at 600 nm [OD_600_] = 0.8), mixed with 2 μL/mL mitomycin C, and incubated for another 2 to 4 h. Phages were harvested by centrifugation for 5 min at 8,150 × *g* and filtering the supernatant with a 0.2-μm membrane filter. The lysogens were obtained as described previously, with slight adjustments (31). In brief, ϕ13kan^R^ was added at a multiplicity of infection (MOI) of 1 to the respective recipients and incubated 30 min on ice to allow phage attachment. The nonattached phages were washed off, and after another incubation for 30 min at 37°C to allow phage infection, the culture was diluted and plated on TSA with 5% blood and 30 μg/mL kanamycin. After overnight incubation at 37°C, 20 colonies showing beta-hemolysis and two colonies without beta-hemolysis were isolated and used for further analysis. Lysogens were derived from eight independent lysogenization experiments resulting in lysogens 1 to 5 (experiment 1), 6 and 7 (experiment 2), 8 (experiment 3), 10 and 11 (experiment 4), 12 and 13 (experiment 5), 14 and 15 (experiment 6), 16 to 19 (experiment 7), and 20 to 23 (experiment 8).

### Spot assay and phage propagation.

Phage lysates were serially diluted in SM-buffer (100 mM NaCl, 50 mM Tris [pH 7.8], 1 mM MgSO_4_, 4 mM CaCl_2_) and spotted on a recipient lawn of S. aureus 8325-4 for PFU determination. To obtain an even lawn, 100 μL of fresh culture (OD = 1) was added to 3 mL top agar and poured on a TSA plate supplemented with 10 mM CaCl_2_. After solidifying of the top agar, three drops of 10 μL each of each dilution were spotted on the lawn.

### Induction assay.

To determine the different levels of phage release, the 8325-4attBmut lysogens were grown to an OD_600_ of 0.8 and centrifuged after addition of 2 μg/mL mitomycin C and further incubation for 2 h. The sterile-filtered supernatant was diluted and spotted on an overlay of 8325-4 consisting of 100 μL culture mixed with 3 mL top agar.

### Whole-genome sequencing and bioinformatics analysis.

Genomic DNA was extracted by using a DNeasy blood and tissue kit (Qiagen), and whole-genome sequences were obtained by 251-bp paired-end sequencing (MiSeq; Illumina) as described previously (32). Genomes were assembled using SPAdes (33). Geneious Prime 2020.1.1 was used to determine phage integration sites. The locations and core sequences were determined by extracting short sequences from the assembled draft genomes of the lysogens lying adjacent to the prophage and mapping it to the annotated genome of S. aureus 8325 (GenBank accession no. NC_007795). Reads obtained by sequencing the PCR amplicons spanning *attP* were mapped to the ϕ13 reference genome (GenBank accession no. NC_004617), and single nucleotide polymorphisms (SNPs) were called by applying a variant frequency threshold of 50%. WebLogo3 was applied to detect gapped motifs in the flanking regions of the alternative *attB* sites (34).

### PCR and amplicon sequencing.

Direct colony PCR was used to determine (i) the presence of the phage using *sak* primers, (ii) the integrity of the *hlb* gene using *hlb* primers, and (iii) *attP* using *attP*st primers (35) if the phage had spontaneously excised and was present in its circular form. Primer sequences and cycling conditions are listed in Table S2. For each reaction, a well-isolated colony was picked, suspended in 50 μL MilliQ water, heat lysed for 5 min at 99°C, and briefly centrifuged. One microliter was used as the template. To determine *attP* of induced phages in lysates, 1 μL of a 1:10 dilution of phage lysate was used as the template. Each single-reaction mixture was composed of 20.375 μL water, 2.5 mL *Taq* polymerase buffer, 1 μL each of forward and reverse primers (10 μM), 0.5 μL deoxynucleoside triphosphates (dNTPs), and 0.125 μL *Taq* polymerase (Thermo Fisher). PCR products were purified with GeneJET PCR purification kit (Thermo Fisher) and sequenced either by Sanger sequencing (Mix2Seq; Eurofins Genomics) for the Sa3int-phages derived from the LA-MRSA strains or by using an Illumina MiSeq system (sequencing depth varied from 10,000 to 180,000 [average, 100,000]).

10.1128/mBio.02259-21.7 TABLE S2 Overview of primers and cycling conditions used for conventional and qPCR. If not otherwise stated, the primers were designed for this study. Download Table S2, PDF file, 0.3 MB. Copyright © 2021 Leinweber et al. 2021 Leinweber et al. https://creativecommons.org/licenses/by/4.0/ This content is distributed under the terms of the Creative Commons Attribution 4.0 International license.

### qPCR assay.

DNA for use in the qPCR assay (LightCycler 96; Roche) was extracted using the GenElute bacterial genomic DNA kit (Sigma). The samples of interest were obtained by lysogenizing S. aureus 8325-4 and 8325-4attBmut with the respective phage (ϕ13kanR, ϕlys2, or ϕlys7) and plating two 100-μL portions of the culture on TSA supplemented with 30 μg/mL kanamycin. After overnight incubation, the colonies were scraped off (approximately 10,000 colonies) and resuspended in 1 mL saline. Of this, 100 μL was used directly in the first lysis step of the kit. DNA concentration was measured using a Qubit fluorometer (Invitrogen) and diluted to 1 ng/mL, of which 5 μL was used in the qPCR, where the reaction mixture consisted of 3 μL water, 10 μL 2× FastStart Essential DNA green master, and 1 μL of each forward and reverse primers (10 μM). Primer sequences and cycling conditions can be found in Table S2.

### Data availability.

All genomic data used or produced in this study have been deposited at the European Nucleotide Archive (https://www.ebi.ac.uk/ena/browser/home). Accession numbers and identifiers are listed in Tables S4 and S5. Source data for the qPCR assay and Sanger amplicon sequencing can be found at https://doi.org/10.17894/ucph.d6a30dc3-54bb-430e-a90c-c4e5baefd3ca with identifiers in Table S4. Raw data can be accessed at https://www.ebi.ac.uk/ena/browser/home with identifiers listed in Table S5 and with BioProject number PRJEB44479.

10.1128/mBio.02259-21.9 TABLE S4 Accession numbers and identifiers for source data of Table S1. The BioProject/BioSample is accessible at https://www.ebi.ac.uk/ena/browser/home. Mix2Seq Sanger sequencing identifiers for PCR fragments containing *attP* of the induced Sa3int-phages; data are available in Fig. 1. The whole sequences of the PCR fragments are deposited at https://doi.org/10.17894/ucph.d6a30dc3-54bb-430e-a90c-c4e5baefd3ca, accessible with the identifiers in Table S4 Table S4, PDF file, 0.2 MB. Copyright © 2021 Leinweber et al. 2021 Leinweber et al. https://creativecommons.org/licenses/by/4.0/ This content is distributed under the terms of the Creative Commons Attribution 4.0 International license.

10.1128/mBio.02259-21.10 TABLE S5 Accession numbers and identifiers for the genomic data generated in this study. Raw reads are available at https://www.ebi.ac.uk/ena/browser/home with BioProject number PRJEB44479. Download Table S5, PDF file, 0.3 MB. Copyright © 2021 Leinweber et al. 2021 Leinweber et al. https://creativecommons.org/licenses/by/4.0/ This content is distributed under the terms of the Creative Commons Attribution 4.0 International license.
