# Supplementary figures and images for: Staphylococcal Phages Adapt to New Hosts by Extensive Attachment Site Variability
Source: mBio. 2021 Dec 7;12(6):e02259-21. doi: 10.1128/mBio.02259-21 (PMC8649754; doi:10.1128/mBio.02259-21)

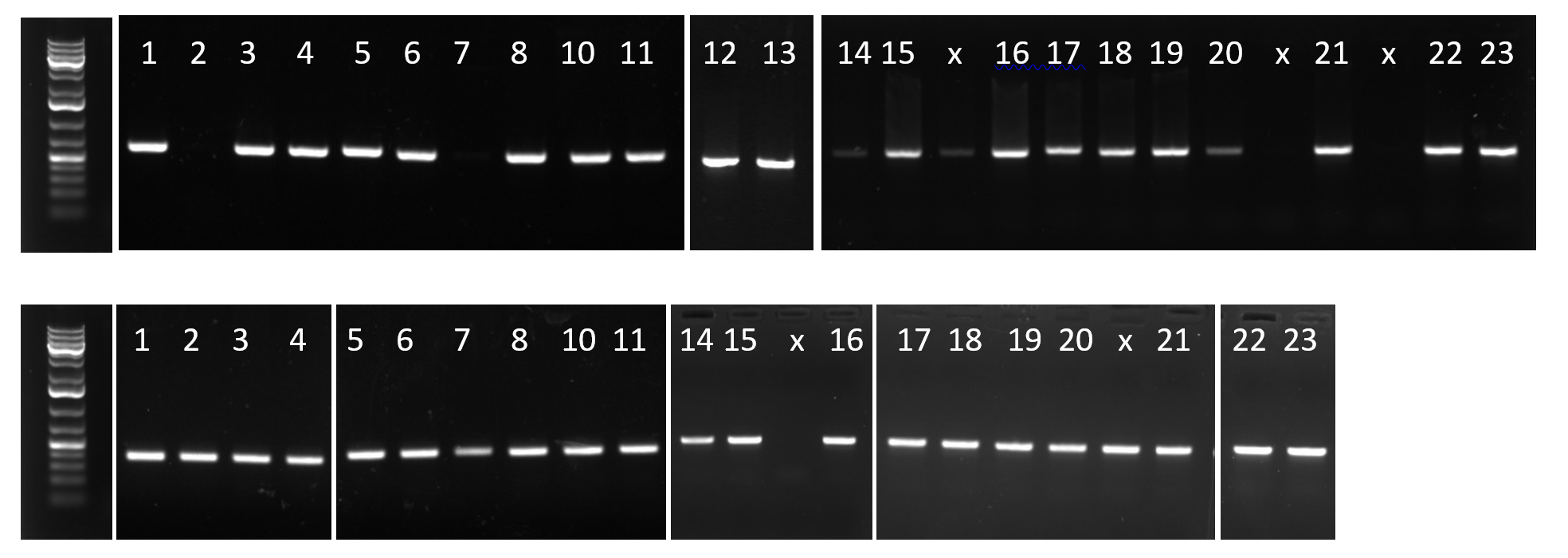

Supplement: FIG S1 [file mbio.02259-21-sf001.tif]

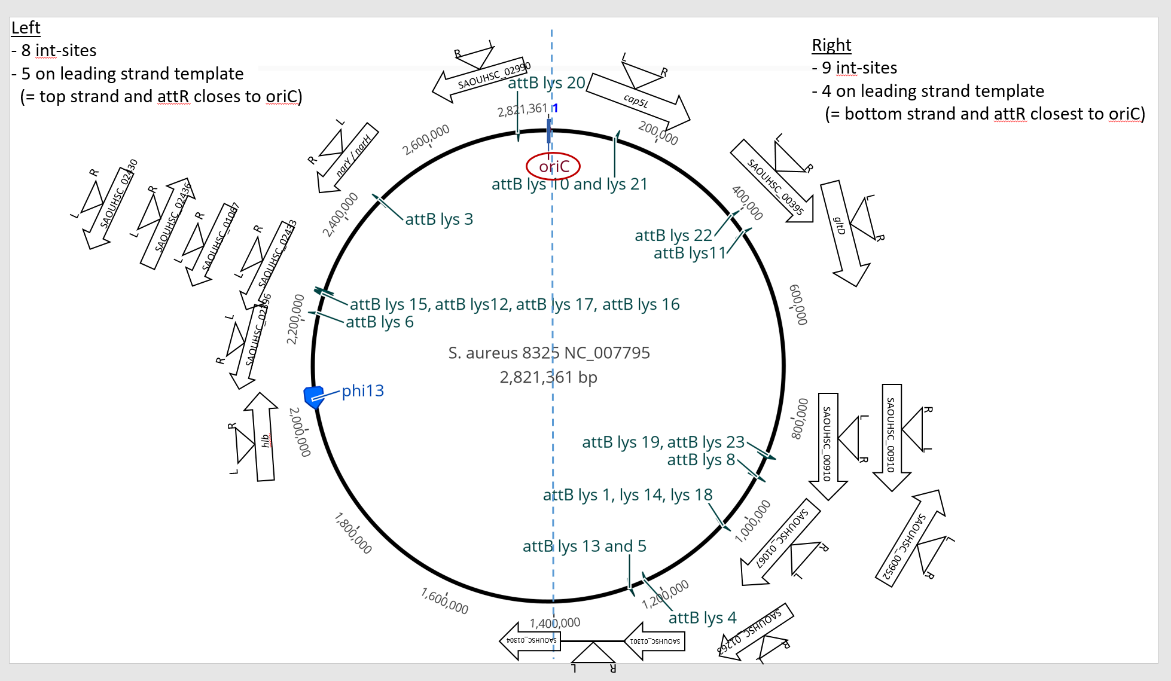

Supplement: FIG S2 [file mbio.02259-21-sf002.tif]

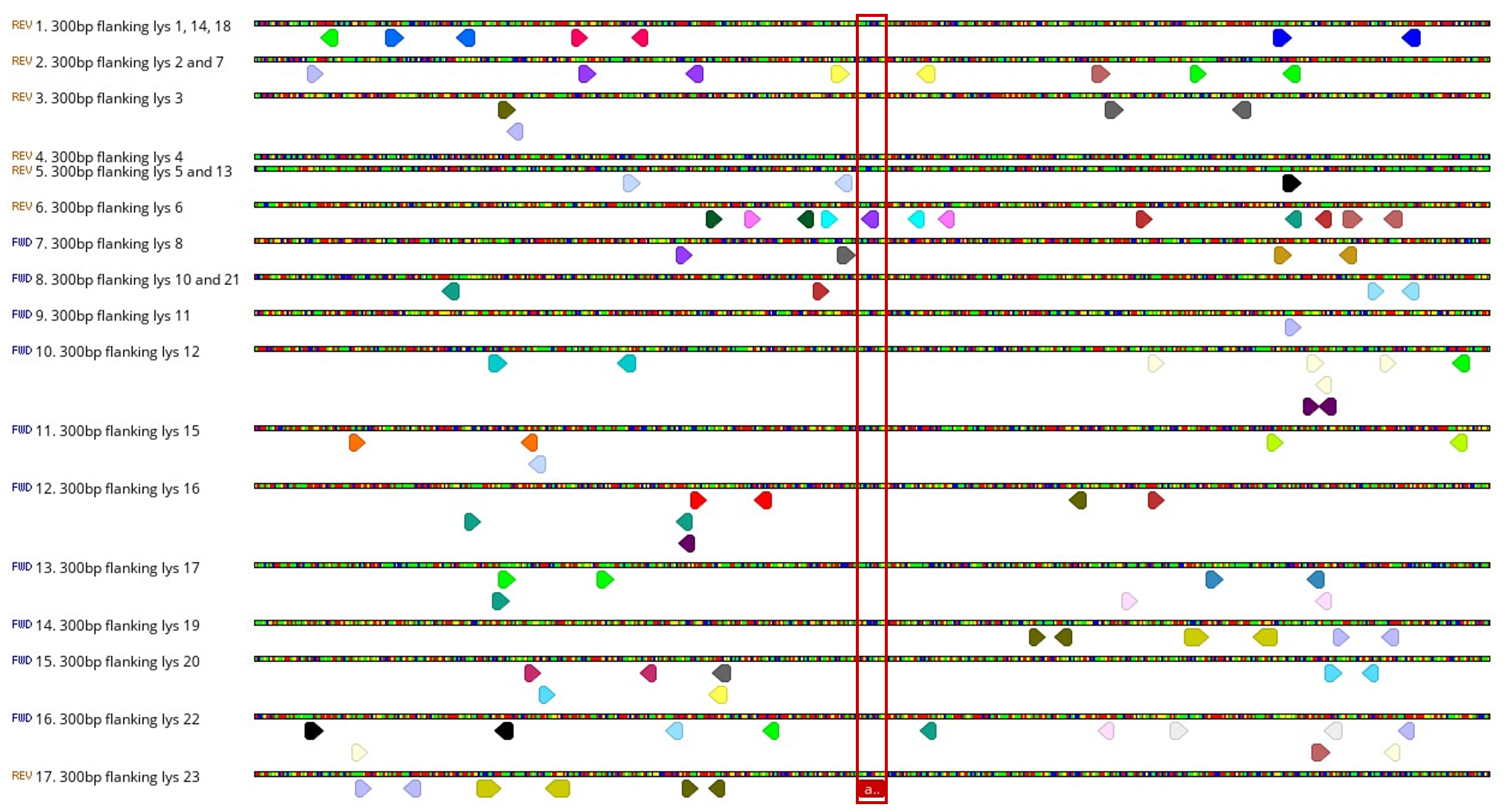

Supplement: FIG S3 [file mbio.02259-21-sf003.tif]

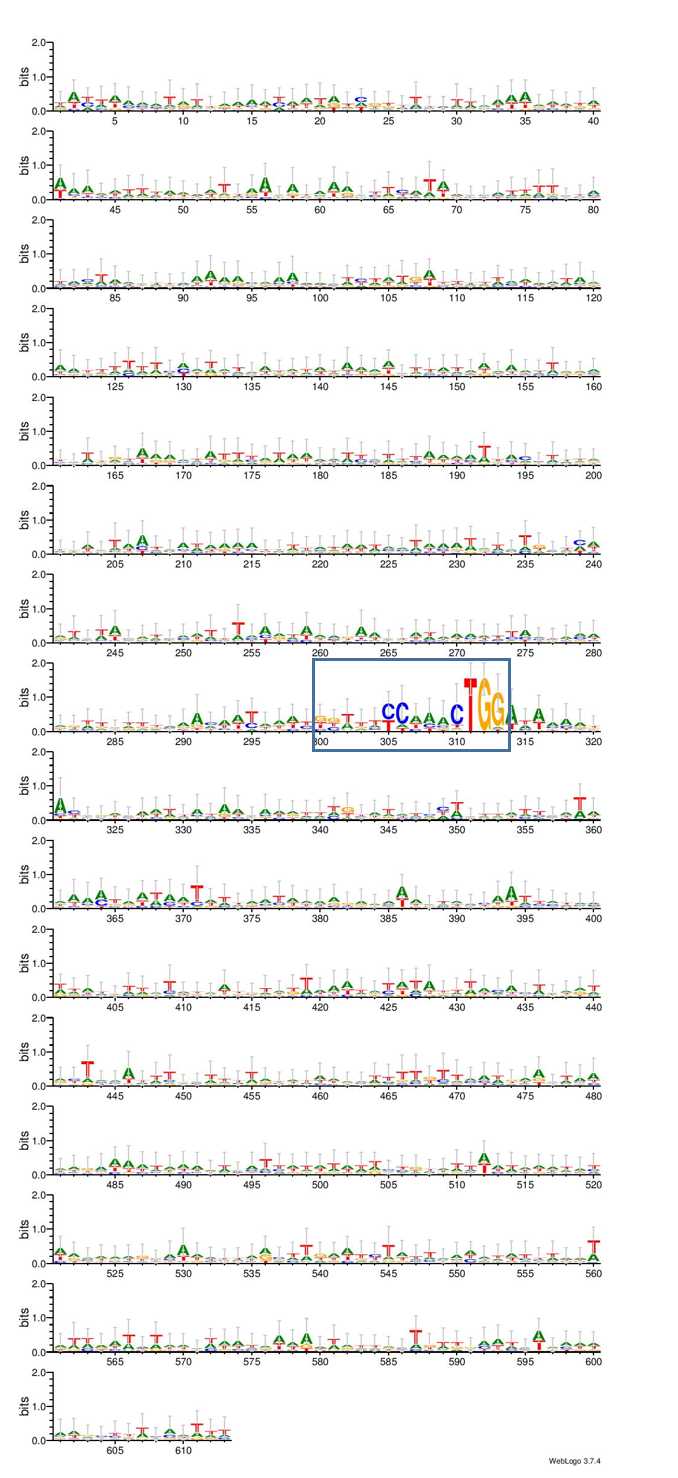

Supplement: FIG S4 [file mbio.02259-21-sf004.tif]

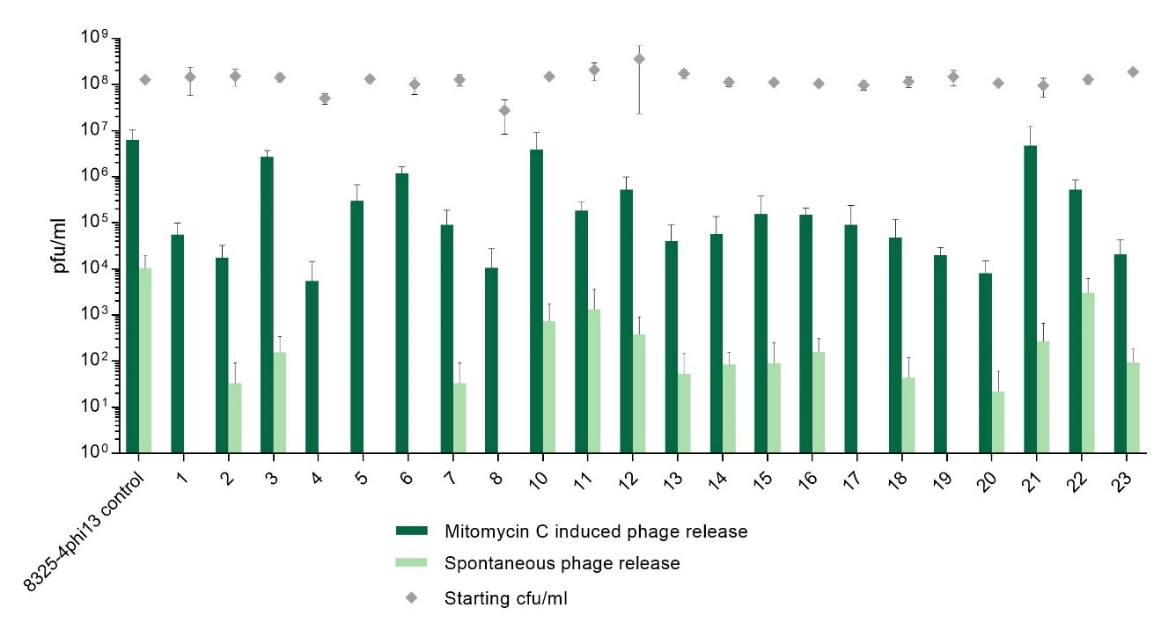

Supplement: FIG S5 [file mbio.02259-21-sf005.tif]
