# Supplementary material for: Staphylococcal Phages Adapt to New Hosts by Extensive Attachment Site Variability
Source: mBio. 2021 Dec 7;12(6):e02259-21. doi: 10.1128/mBio.02259-21 (PMC8649754; doi:10.1128/mBio.02259-21)
Supplement: TABLE S3 [file mbio.02259-21-st003.pdf]

Alternative *attB*	Location	Reference genome	Accession no.	Ref.
TGTATCCAAACTGG	Original *attB* in *hlb* of *S. aureus* 8325-4			
GTTATCCAATCTGG	alanine racemase	*S. aureus* CC398, 61599	PRJEB25608	(4)
GGGGACCTAACTGG	nitrate reductase			
CCATTCCATACTGG	ornithine carbamoyltrasferase			
GTGTATCCATCTGG	FADH(2)-oxidizing methlentetrahydrofolate-tRNA-(uracil(54)-C(5))-methyltransferase TrmFO)			
TTTATCGTTTCTGG	Acycl ersterase			
TTTATCCGTAATGC	between SAPIG2164: aldehyde dehydrogenase family protein and SPIG2165: HxlR family transcriptional regulator			
TGTTCTTTATCTGG	plasmid JQ861959 integrated upstream of the *muTB* gene			
GTTTCTCCACCTGG	membrane protein (2x)			
CGATGCCAAAATGG	pyruvate oxigenase	*S. aureus* ST398	AM990992.1	(4)
TGTACCTTATGAGG	*ilvB*			
CTAGTCCTTACTGT	between SAPIG0723 and SAPIG0724 (2x, different *attR* and *attL*, indicating different *attP*)			
GTTATCCAATCTGG	alanine racemase			
TGTATCCGAATTGG	*hlb* (10 isolates where *attR* and *attL* are identical to *attB*$_{LA}$, 5 isolates where *attL*=*attB*$_{LA}$ and attR=attB, indicating different *attP*)			
TGATTCCAACCGGG	Hypothetical protein StauST398-5_0050	phage StauST398-5	KC595279	
TGTATCCGAATTGG	*hlb*		CP019593	(7)
CCTTTCCATAATGG	Alpha/beta hydrolase		CP040229	
ACTTCCCAAACTGG	cytochrome d ubiquinol oxidase subunit II		CP040232	
TGTATCCAAACTGG	General stress protein (AUC50_04635)	*S. aureus* RIVM3897	CP013621	(8)
TGTATCCAATCTGG	General stress protein (AUC50_04635)			
TGTATCCTTACTGT	Nucleoside permease (AUC50_03495)			
TGTATCCAAACTGG	Cytochrome D ubiquinol oxidase subunit I (AUC50_05470)			
TGTATCCAAACTGA	Hypothetical protein (AUC50_10740)			
TGTATCCAAACTGA	GMP synthetase (AUC50_02200)			
TGTATCCAAGCTGG	Gamma-aminobutyrate permease (AUC50_08995) (3x)			
TGTATCCAAACTGG	Hypothetical protein (SAMI_1999)	*S. aureus* MI	AP017320	
TGTATCCATACTGG	Alanine racemase (A7327_11665)	*S. aureus* 08–02300	CP015646	
TGTATCCAAACTGG	Integrase	*S. aureus* Sa54	KT253891	

n. a.	ORF SA1270<br>intergenic between ORF SA0966 and SA0967<br>*capL*	*S. aureus* N315	BA000018.3	(9)

4. van Alen S, Ballhausen B, Kaspar U, Köck R. 2018. Prevalence and Genomic Structure of Bacteriophage phi3 in Human-Derived Livestock-Associated Methicillin-Resistant Staphylococcus aureus Isolates from 2000 to 2015. J Clin Microbiol 56:1–11.

7. Kashif A, McClure J-A, Lakhundi S, Pham M, Chen S, Conly JM, Zhang K. 2019. Staphylococcus aureus ST398 Virulence Is Associated With Factors Carried on Prophage φSa3. Front Microbiol.

8. Kraushaar B, Hammerl JA, Kienöl M, Heinig ML, Sperling N, Thanh MDi, Reetz J, Jäckel C, Fetsch A, Hertwig S. 2017. Acquisition of virulence factors in livestock-associated MRSA: Lysogenic conversion of CC398 strains by virulence gene-containing phages. Sci Rep 7:1–13.

9. Goerke C, Koller J, Wolz C. 2006. Ciprofloxacin and trimethoprim cause phage induction and virulence modulation in Staphylococcus aureus. Antimicrob Agents Chemother 50:171–177.
