# Supplementary material for: Staphylococcal Phages Adapt to New Hosts by Extensive Attachment Site Variability
Source: mBio. 2021 Dec 7;12(6):e02259-21. doi: 10.1128/mBio.02259-21 (PMC8649754; doi:10.1128/mBio.02259-21)
Supplement: TABLE S1 [file mbio.02259-21-st001.pdf]

Referred to in this study	Other name	Description	Reference
8325-4		phage cured NCTC8325	(1)
8325-4attBmut	8325-4ϕ13attBmut	8325-4 mutated at ϕ13 attB site in hlb, hlb+	(2)
8325-4ϕ13kan^R		8325-4 lysogenized with ϕ13kanR, hlb-, kanamycin resistant	(2)
Variant I	123193	CC398, Sa3int phage intergenic between SAPIG_RS03725 and SAPIG_RS03730	(3)
Variant I	146281	CC398, Sa3int phage intergenic between SAPIG_RS03725 and SAPIG_RS03730	(3)
Variant I	147327	CC398, Sa3int phage intergenic between SAPIG_RS03725 and SAPIG_RS03730	(3)
Variant I	147333	CC398, Sa3int phage intergenic between SAPIG_RS03725 and SAPIG_RS03730	(3)
Variant I	147335	CC398, Sa3int phage intergenic between SAPIG_RS03725 and SAPIG_RS03730	(3)
Variant II	147331	CC398, Sa3int phage in sfaC SAPIG_RS11745	(3)
Variant II	147339	CC398, Sa3int phage in sfaC SAPIG_RS11745	(3)
Variant III	157445	CC398, Sa3int phage in sph SAPIG_RS10795	(3)
Variant III	157659	CC398, Sa3int phage in sph SAPIG_RS10795	(3)
Variant III	157661	CC398, Sa3int phage in sph SAPIG_RS10795	(3)
Variant III	157663	CC398, Sa3int phage in sph SAPIG_RS10795	(3)
Variant IV	155519	CC398, Sa3int phage in cidA SAPIG_RS13630	(3)
Variant V	153891	CC398, Sa3int phage in sph SAPIG_RS10795	(3)
Variant VI	154501	CC398, Sa3int phage in CocE/NonD SAPIG_RS13905	(3)
Variant VI	154789	CC398, Sa3int phage in CocE/NonD SAPIG_RS13905	(3)
Variant VI	154791	CC398, Sa3int phage in CocE/NonD SAPIG_RS13905	(3)
Variant VI	154923	CC398, Sa3int phage in CocE/NonD SAPIG_RS13905	(3)
Variant VI	55-103-045	CC398, Sa3int phage in CocE/NonD SAPIG_RS13905	(3)
Variant VI	55-103-046	CC398, Sa3int phage in CocE/NonD SAPIG_RS13905	(3)
Variant VI	55-103-047	CC398, Sa3int phage in CocE/NonD SAPIG_RS13905	(3)
Lysogen 1		8325-4attBmut with ϕ13kan^R in SAOUHSC_01067 CDS conserved hypothetical protein	this study
Lysogen 2		8325-4attBmut with ϕ13kan^R in hlb sphingomyelin phosphodiesterase	this study
Lysogen 3		8325-4attBmut with ϕ13kan^R in SAOUHSC_02680 narY nitrate reductase subunit beta	this study
Lysogen 4		8325-4attBmut with ϕ13kan^R inSAOUHSC_01263 phosphodiesterase	this study
Lysogen 5		8325-4attBmut with ϕ13kan^R intergenic between hypothetical proteins SAOUHSC_01301 and SAOUHSC_01304	this study
Lysogen 6		8325-4attBmut with ϕ13kan^R in SAOUHSC_02396 hypothetical protein	this study
Lysogen 7		8325-4attBmut with ϕ13kan^R in hlb sphingomyelin phosphodiesterase	this study

Lysogen 8		8325-4attBmut with ϕ13kan^R in SAOUHSC_00952 hypothetical protein	this study
Lysogen 10		8325-4attBmut with ϕ13kan^R in SAOUHSC_00125 cap5L protein/glycosyltransferase	this study
Lysogen 11		8325-4attBmut with ϕ13kan^R in SAOUHSC_00436 gltD glutamate synthase subunit beta	this study
Lysogen 12		8325-4attBmut with ϕ13kan^R in SAOUHSC_02433 hypothetical protein	this study
Lysogen 13		8325-4attBmut with ϕ13kan^R intergenic between hypothetical proteins SAOUHSC_01301 and SAOUHSC_01304	this study
Lysogen 14		8325-4attBmut with ϕ13kan^R in SAOUHSC_01067 CDS conserved hypothetical protein	this study
Lysogen 15		8325-4attBmut with ϕ13kan^R in SAOUHSC_02428 hypothetical protein	this study
Lysogen 16		8325-4attBmut with ϕ13kan^R in SAOUHSC_02436 hypothetical protein	this study
Lysogen 17		8325-4attBmut with ϕ13kan^R in SAOUHSC_02430 ABC transporter substrate-binding protein CDS	this study
Lysogen 18		8325-4attBmut with ϕ13kan^R in SAOUHSC_01067 CDS conserved hypothetical protein	this study
Lysogen 19		8325-4attBmut with ϕ13kan^R in SAOUHSC_00910 hypothetical protein CDS	this study
Lysogen 20		8325-4attBmut with ϕ13kan^R in SAOUHSC_02990 hypothetical protein CDS	this study
Lysogen 21		8325-4attBmut with ϕ13kan^R in SAOUHSC_00125 cap5L protein/glycosyltransferase	this study
Lysogen 22		8325-4attBmut with ϕ13kan^R in SAOUHSC_00395 superantigen-like protein CDS	this study
Lysogen 23		8325-4attBmut with ϕ13kan^R in SAOUHSC_00910 hypothetical protein CDS	this study

1. Novick R. 1967. Properties of a cryptic high-frequency transducing phage in Staphylococcus aureus. Virology 33:155–166.

2. Tang Y, Nielsen LN, Hvitved A, Haaber JK, Wirtz C, Andersen PS, Larsen J, Wolz C, Ingmer H. 2017. Commercial biocides induce transfer of prophage Φ13 from human strains of Staphylococcus aureus to livestock CC398. Front Microbiol 8:1–11.

3. Sieber RN, Urth TR, Petersen A, Møller CH, Price LB, Skov RL, Larsen AR, Stegger M, Larsen J. 2020. Phage-mediated immune evasion and transmission of livestock-associated methicillin-resistant staphylococcus aureus in humans. Emerg Infect Dis 26:2578–2585.
