# Supplementary material for: Staphylococcal Phages Adapt to New Hosts by Extensive Attachment Site Variability
Source: mBio. 2021 Dec 7;12(6):e02259-21. doi: 10.1128/mBio.02259-21 (PMC8649754; doi:10.1128/mBio.02259-21)
Supplement: TABLE S2 [file mbio.02259-21-st002.pdf]

Primer name	*Sequence 5´- 3´*	*Annealing temp. °C*	*Elongation time (s)*	*Reference*
hlb_fwd	ATGGTGAAAAAAACAAAATCCAATTCAC	50	60	This study
hlb_rev	CTATTTACTATAGGCTTTGATTGGGTAATG			
Sak_fwd	GTGCATCAAGTTCATTCGAC	49	30	(4)
Sak_rev	TAAGTTGAATCCAGGGTTTT			
attPst_fwd	TCTAGCTTTTGGGGTGTACATTCC	49	30	(5)
attPst_rev	GCTTTGAAATCAGCCTGTAGAG			
Pta fwd	AGAAGCAATCATTGATGGCGA	55	10	(6)
Pta rev	ACCTGGCGCTTTTTTCTCAG			
Φ13 attR fwd	CTCCAAACCCAATAAATACTGTTGTTAC	55	10	This study
Hlb attR rev	CGAGTACAGGTGTTTGATAAGGATATTC	55	10	This study

4. van Alen S, Ballhausen B, Kaspar U, Köck R. 2018. Prevalence and Genomic Structure of Bacteriophage phi3 in Human-Derived Livestock-Associated Methicillin-Resistant Staphylococcus aureus Isolates from 2000 to 2015. J Clin Microbiol 56:1–11.

5. Goerke C, Wirtz C, Flückiger U, Wolz C. 2006. Extensive phage dynamics in Staphylococcus aureus contributes to adaptation to the human host during infection. Mol Microbiol 61:1673–1685.

6. Aedo S, Tomasz A. 2016. Role of the stringent stress response in the antibiotic resistance phenotype of methicillin-resistant staphylococcus aureus. Antimicrob Agents Chemother 60:2311–2317.
