# Supplementary material for: Staphylococcal Phages Adapt to New Hosts by Extensive Attachment Site Variability
Source: mBio. 2021 Dec 7;12(6):e02259-21. doi: 10.1128/mBio.02259-21 (PMC8649754; doi:10.1128/mBio.02259-21)
Supplement: TABLE S4 [file mbio.02259-21-st004.pdf]

Name used in this study	Study ID	Other ID	Bioproject	Biosample	Mix2Seq number
Variant I	123193	SSI_123193	PRJEB25608	ERR1992266	EF30859333
Variant I	146281	SSI_146281	PRJNA613886	SRR11364549	EF30859334
Variant I	147327	SSI_147327	PRJNA613886	SRR11364546	EF30859336
Variant I	147333	SSI_147333	PRJNA613886	SRR11364544	EF30859338
Variant I	147335	SSI_147335	PRJNA613886	SRR11364543	EF31211630
Variant II	147331	SSI_147331	PRJNA613886	SRR11364545	EF30859337
Variant II	147339	SSI_147339	PRJNA613886	SRR11364542	EF31211645
Variant III	157445	SSI_157445	PRJNA613886	SRR11364487	EF31634870
Variant III	157659	SSI_157659	PRJNA613886	SRR11364554	EF31634871
Variant III	157661	SSI_157661	PRJNA613886	SRR11364553	EF31634872
Variant III	157663	SSI_157663	PRJNA613886	SRR11364552	EF31634874
Variant IV	155519	SSI_155519	PRJNA613886	SRR11364507	EF31634869
Variant V	153891	SSI_153891	PRJNA613886	SRR11364516	EF31211647
Variant VI	154501	SSI_154501	PRJNA613886	SRR11364513	EF31634868
Variant VI	154789	SSI_154789	PRJNA613886	SRR11364512	EF31211648
Variant VI	154791	SSI_154791	PRJNA613886	SRR11364511	EF31203716
Variant VI	154923	SSI_154923	PRJNA613886	SRR11364510	EF31888721
Variant VI	55-103-045		PRJEB25608	ERR1992132	EF31211652
Variant VI	55-103-046		PRJEB25608	ERR1992133	EF31211815
Variant VI	55-103-047		PRJEB25608	ERR1992134	EF31211656
