# Supplementary material for: Staphylococcal Phages Adapt to New Hosts by Extensive Attachment Site Variability
Source: mBio. 2021 Dec 7;12(6):e02259-21. doi: 10.1128/mBio.02259-21 (PMC8649754; doi:10.1128/mBio.02259-21)
Supplement: TABLE S5 [file mbio.02259-21-st005.pdf]

Name used in this study	Biosample
Lysogen 1	SAMEA8606235
Lysogen 2	SAMEA8606236
Lysogen 3	SAMEA8606237
Lysogen 4	SAMEA8606238
Lysogen 5	SAMEA8606239
Lysogen 6	SAMEA8606240
Lysogen 7	SAMEA8606229
Lysogen 8	SAMEA8606230
Lysogen 10	SAMEA8606231
Lysogen 11	SAMEA8606232
Lysogen 12	SAMEA8606233
Lysogen 13	SAMEA8606234
Lysogen 14	SAMEA8606241
Lysogen 15	SAMEA8606242
Lysogen 16	SAMEA8606243
Lysogen 17	SAMEA8606244
Lysogen 18	SAMEA8606245
Lysogen 19	SAMEA8606246
Lysogen 20	SAMEA8606247
Lysogen 21	SAMEA8606248
Lysogen 22	SAMEA8606249
Lysogen 23	SAMEA8606250
attP Φlys1	SAMEA8606266
attP Φlys2	SAMEA8606267
attP Φlys3	SAMEA8606251
attP Φlys4	SAMEA8606268
attP Φlys5	SAMEA8606253
attP Φlys6	SAMEA8606255
attP Φlys7	SAMEA8606257
attP Φlys8	SAMEA8606259
attP Φlys10	SAMEA8606269
attP Φlys11	SAMEA8606261
attP Φlys12	SAMEA8606270
attP Φlys13	SAMEA8606263
attP Φlys14	SAMEA8606265
attP Φlys15	SAMEA8606271
attP Φlys16	SAMEA8606252
attP Φlys17	SAMEA8606254
attP Φlys18	SAMEA8606256

attP Φlys19	SAMEA8606258
attP Φlys20	SAMEA8606260
attP Φlys21	SAMEA8606262
attP Φlys22	SAMEA8606264
attP Φlys23	SAMEA8606272
attP Φphi13kan^R	SAMEA8606273
